# Circulating Lipids Are Associated with Alcoholic Liver Cirrhosis and Represent Potential Biomarkers for Risk Assessment

**DOI:** 10.1371/journal.pone.0130346

**Published:** 2015-06-24

**Authors:** Peter J. Meikle, Piyushkumar A. Mundra, Gerard Wong, Khairunnessa Rahman, Kevin Huynh, Christopher K. Barlow, Alastair M. P. Duly, Paul S. Haber, John B. Whitfield, Devanshi Seth

**Affiliations:** 1 Baker IDI Heart and Diabetes Institute, Melbourne, Vic 3004, Australia; 2 Discipline of Clinical Medicine & Addiction Medicine, Faculty of Medicine, University of Sydney, Sydney, NSW 2006, Australia; 3 Garvan Institute of Medical Research, Darlinghurst, NSW 2010, Australia; 4 Drug Health Services and Centenary Institute of Cancer Medicine and Cell Biology, Royal Prince Alfred Hospital, Camperdown, NSW 2050, Australia; 5 Genetic Epidemiology, QIMR Berghofer Medical Research Institute, Herston, Queensland 4029, Australia; Institute of Medical Research A Lanari-IDIM, University of Buenos Aires-National Council of Scientific and Technological Research (CONICET), ARGENTINA

## Abstract

Liver disease is the greatest cause of death related to alcohol and a major public health problem. While excessive alcohol intake results in hepatosteatosis in most individuals, this can progress in some to more severe forms of liver disease including fibrosis and cirrhosis. An ongoing challenge in the management of alcoholic liver disease is the identification of liver injury early in the disease process such that intervention strategies can prevent serious long term outcomes. Given that excessive alcohol consumption results in dysregulation of lipid metabolism we applied lipid profiling technology to characterise and compare serum lipid profiles from excessive chronic drinkers with no liver disease to those with advanced alcoholic cirrhosis. In a cohort of 59 excessive drinkers (31 with liver cirrhosis and 28 with no evidence of liver disease) we used electrospray ionisation tandem mass spectrometry to measure over 300 individual lipid species in serum, including species of the major phospholipid, sphingolipid, glycerolipid and sterol classes. Six of the 25 lipid classes and subclasses were significantly associated with alcoholic liver cirrhosis; these included dihexosylceramide, trihexosylceramide, alkylphosphatidylcholine, lysoalkylphosphatidylcholine, phosphatidylinositol and free cholesterol. Multivariate classification models created with only clinical characteristics gave an optimal model with an AUC of 0.847 and an accuracy of 79.7%. The addition of lipid measurements to the clinical characteristics resulted in models of improved performance with an AUC of 0.892 and accuracy of 81.8%. The gain in AUC and accuracy of the combined models highlight the potential of serum lipids as markers of liver injury in alcoholic liver disease.

## Introduction

The excessive use of alcohol is widely recognised as a major health and social problem worldwide. Of the 60 medical diseases associated with alcohol, alcoholic liver disease causes the most deaths and is consequently the greatest public health problem. Excessive alcohol consumption, defined as more than 4/3 drinks on any single day or more than 14/7 drinks per week (for men/women) [1], leads to hepatosteatosis or fatty liver in more than 90% of drinkers, which is reversible on abstinence. Nonetheless, continued drinking can lead to progressive liver injury. Moreover, alcohol itself can promote carcinogenesis through lipid peroxide generation implicated in alcohol-related hepatocarcinogenesis [2].

A key issue with alcoholic liver cirrhosis (ALC) is the early identification of liver injury. The ability to distinguish between mild inflammation and progressive severe liver disease would enable the targeting of intensive efforts to reduce alcohol consumption and/or guide specific treatment to arrest disease progression. Standard clinical tests (blood tests, ultrasound and computed tomography scanning) perform poorly in this regard due to lack of both sensitivity and specificity. Currently available biomarkers rely on alcohol metabolic products (alcohol consumption markers) or liver injury (non-ALC specific markers). There is a need to identify new biomarkers specific for the early diagnosis of ALC, distinct from biomarkers of alcohol consumption.

Dyslipidemia is a common feature of alcoholic cirrhosis. One of the known mechanisms of alcohol induced liver injury is via alteration of lipid processing pathways, including fatty acid synthesis, uptake, oxidation and export from the liver [3,4]. Tracer studies in animals [5] and humans [6] have also demonstrated an alteration in de-novo lipogenesis resulting from alcohol consumption leading to altered liver and plasma lipid concentrations. In mouse studies, chronic alcohol consumption was associated with increased hepatic free fatty acid and decreased acyl-CoA levels leading to increased levels of ceramide and the ceramide metabolites sphingosine and sphinganine [7]. Zhao *et al*. reported ethanol-induced tissue-specific changes in lipids related to specific fatty acid species in tissues and plasma [8].

In common with alcoholic steatosis, the elevated free fatty acids associated with obesity contribute to the progression to type 2 diabetes. Insulin, an anti-lipolytic hormone, decreases mitochondrial β-oxidation of both free and hepatic fatty acids and thus increases hepatic fat accumulation. Insulin resistance can therefore induce steatosis by favouring peripheral lipolysis and hepatic lipid accumulation. Indeed, hepatic fat was inversely related to insulin sensitivity in a study including insulin-resistant and insulin-sensitive men [9]. Exogenous insulin administered to treat elevated blood glucose was more effective in patients with less liver fat [10]. Finally, the effects of insulin-sensitizing drugs rosiglitazone [11] and pioglitazone [12] include reversal of steatosis, suggesting that to some extent steatosis was responsible for insulin resistance. Recent cohort studies showed that obese drinkers are more prone to developing cirrhosis than drinkers in a healthy weight range [13,14] suggesting an interaction in the pathobiology of ALC, obesity and insulin sensitivity. We recently characterised the effect of elevated free fatty acids on circulating lipids in the settings of obesity, prediabetes and type 2 diabetes using a lipidomic approach [15,16]. Multiple lipid metabolic pathways were affected leading to alterations of the circulating levels of specific sphingolipid, phospholipid and glycerolipid species. In this study we characterised serum lipid profiles from excessive chronic drinkers with no liver disease (Controls) to those with advanced alcoholic liver cirrhosis (Cases).

## Materials and Methods

### Patient details

Recruitment of excessive drinkers from alcohol treatment centres and liver clinics was conducted at the four major hospitals (Royal Prince Alfred, Concord Repatriation General, Liverpool and Fairfield) in Sydney, Australia. Written informed consent was obtained from all participants. The study was approved by the Royal Prince Alfred Hospital Ethics Committee (HREC/11/RPAH/88).

Excessive drinkers are defined as having daily alcohol consumption of ≥80 g per day (men) and 50 g per day (women) for ten years or more. Cases (n = 31) have clinical and/or histopathology evidence of ALC, with exclusion of hepatitis B or C, autoimmune liver disease, haemochromatosis and Wilson’s Disease (S1 Table). Controls (n = 28) have normal results for AST, ALT, total bilirubin, albumin, platelet count and INR while actively drinking or within 7 days of stopping the most recent episode of heavy alcohol use and no clinical and/or biopsy evidence of significant liver disease (S1 Table). HIV infection is an exclusion criterion for both Cases and Controls.

Clinical data from consenting participants included alcohol and tobacco history (amount, type, duration, abstinence, parental history), other drug use, demographics (age, gender, ethnicity, education, marital status, medication), blood biochemistry (liver function tests, bilirubin, blood clotting by international normalised ratio (INR), creatinine), clinical evidence of liver injury (ascites, encephalopathy, variceal bleeding), anthropometrics and Alcohol Use Disorder Identification Test [17]. Patient characteristics for the two groups are presented in Table 1. Serum was prepared from non-fasting blood and stored at -80°C. The majority of the samples were non-fasting due to the unpredictable nature of patients presenting at the drug treatment centre, that did not allow strict regimens of blood collection times and fasting status.

**Table 1:** Patient characteristics.

Characteristics	Control (Median and IQR^1^) (n = 28)	Case (Median and IQR^1^) (n = 31)	p-value^2^
Age (year)	47.0 (41.5–56.0)	59.0 (54.0–60.8)	**0.0033**
Sex (F/M)^3^	4/24	5/26	0.94
Body Mass Index (Kg/m^2^)	24.0 (22.4–25.5)	26.9 (23.2–31.0)	**0.0148**
Systolic Blood Pressure (mm Hg)	126 (120–135)	126 (118–138)	0.62
Diastolic Blood Pressure (mm Hg)	80 (75–90)	79 (72–83)	0.19
Alanine Transaminase (IU/L)	29.5 (20.0–52.0)	28.0 (21.8–35.8)	0.99
Aspartate Transaminase (IU/L)	31.5 (23.0–50.5)	46.0 (33.0–54.8)	**0.0223**
Total Bilirubin (μmol/L)	9.0 (6.0–13.5)	29.0 (12.5–51.3)	**1.14E-05**
GGT (IU/L)	110 (56–184)	101 (39–145)	0.62
AST/ALT^4^	1.05 (0.86–1.39)	1.57 (1.05–1.99)	**0.0067**
MELD^5^ score	6.43 (5.25–7.50)	13.89 (9.51–17.52)	**3.18E-09**
Creatinine (μmol/L)	73.0 (58.0–80.5)	71.0 (59.5–99.8)	0.33
INR^6^	1.00 (0.90–1.00)	1.30 (1.10–1.50)	**1.70 E-07**
Albumin (g/L)	45.5 (41.5–47.0)	35.0 (31.0–40.8)	**9.69E-06**
Triglyceride (mg/dL)	124 (89–204)	106(71–159)	0.89
Total Chol (mg/dL)	186 (159–213)	170 (157–217)	0.10
LDL (mg/dL)	89 (77–120)	101 (77–137)	0.28
HDL (mg/dL)	60 (50–75)	52 (44–65)	0.07
Hemoglobin (g/L)	145 (135–157)	116 (106–126)	**1.42E-06**
White Blood Cell Count	6.40 (5.55–9.30)	5.10 (3.63–7.05)	**0.0088**
Platelet Count	259 (205–334)	101 (64–151)	**3.00E-07**
Alcohol (g/day)	200 (150–250)	180 (120–240)	0.19
Alcohol (kg/year)	73.0 (54.8–92.7)	65.7 (43.8–87.6)	0.19
Alcohol (kg/lifetime)	1387 (853–1701)	1139 (827–1982)	0.80

^**1**^ IQR, interquartile range.

^2^ Unless otherwise stated, all p-values are computed using the Mann-Whitney U test.

^3^ p-value is computed using the chi-square test.

^4^ Aspartate transaminase/alanine aminotransferase

^5^ Model for end-stage liver disease.

^6^ International Normalized Ratio for prothrombin time.

### Lipid extraction

Lipids were extracted from serum as described previously [16]. In brief, 200μL of a 2:1 choloroform:methanol mix, together with 10 μL of the internal standard mix was added to 10μL serum. The samples were vortexed for 10mins, incubated in an ultrasonic water bath at 20°C for 30mins and rested for 20mins at ambient temperature. After centrifuging the samples (13,000 g, 10mins) supernatants containing extracted lipids were dried under N_2_. The dried lipids were resuspended in 50μL water-saturated butanol and 50μL 10mM NH_4_COOH in methanol. After centrifuging (4000 g, 5mins) supernatants were transferred into HPLC glass vials with 0.2mL micro-inserts and Teflon insert caps.

### Lipidomic analysis

Liquid chromatography electrospray ionisation tandem mass spectrometry was conducted as previously described [16]. Briefly, online LC-MS/MS was performed using a Zorbax C18, 1.8 μm, 50 x 2.1 mm column (Agilent Technologies) connected to the ionization source of an API4000 QTRAP mass spectrometer (Applied Biosystems). Liquid chromatography was performed using the following gradient conditions; 0–100% buffer B over 8.0 min, 2.5 min at 100% B, a return to 0% B over 0.5 min then 3.0 min at 0% B prior to the next injection. Diacylglycerols and triacylglycerols were separated using the same solvent system with an isocratic flow (100 μL/min) of 85% B. Solvents A and B consisted of tetrahydrofuran:methanol:water (30:20:50 and 75:20:5 respectively), both containing 10 mM NH_4_COOH. Measurement of individual lipid species was performed using scheduled multiple-reaction monitoring in positive ion mode. Lipid measures were calculated by relating the peak area of each species to the peak area of the corresponding internal standard. Cholesteryl ester species were corrected for response factors determined for each species. Total measured lipids of each class were calculated by summing the individual lipid species.

### Statistical Analysis

Prior to regression analysis, data for each lipid species, class or subclass was standardised to the interquartile range (IQR). This facilitates the comparison of odds ratios or beta coefficients between lipid species with varying concentration ranges. Binary logistic regression analysis adjusted for age and BMI was performed to determine the association of lipid classes, subclasses and individual lipid species with ALC case-control status. The resultant IQR odds ratio for a given lipid measurement represents the number of times an individual with a lipid measurement at the 75^th^ percentile is more likely to have ALC than an individual with a lipid measurement at the 25^th^ percentile. Linear regression adjusted for age and BMI was used to determine the linear relationship between lipids and the model for end stage liver disease (MELD) score or bilirubin level. The beta-coefficients obtained represent the change in outcome measure (MELD score or bilirubin level) associated with an interquartile range increase in the lipid measurement. For all analyses, p-values were corrected for multiple comparisons using the Benjamini-Hochberg method [18] and statistical significance was determined by a corrected p-value <0.05.

The classification performance of lipid species and clinical characteristics were evaluated using machine learning methods. Along with lipid species, the following clinical characteristics were used: sex, BMI, systolic blood pressure, diastolic blood pressure, white blood count, haemoglobin, platelet count, albumin, bilirubin, creatinine, INR, aspartate aminotransferase to alanine aminotransferase ratio, gamma-glutamyl transferase, cholesterol, triglyceride, LDL-C, HDL-C, daily, yearly, and life time alcohol consumption. As age was significantly different between the ALC and alcoholic without liver cirrhosis (AwLC) groups, all the lipid species measurements and clinical characteristic values were adjusted for age based on the residuals from linear regression. After thresholding all the negative values to one, all the adjusted values were log-transformed.

To classify ALC from AwLC, we performed feature selection, model training and testing within a 3-fold stratified cross-validation framework (repeated 200 times). In each iteration, the order of inclusion of features into the model was determined by the reliefF algorithm [19]. Model development involved the implementation of a linear kernel-based support vector machine classifier obtained from the libraries of LIBSVM 2.84 [20]. The performance the model was assessed on the test dataset by computing the C-statistic (Area Under the Curve (AUC)), accuracy, specificity, sensitivity and net reclassification index for the first 30 features included in the model. Finally, the mean and 95% confidence intervals of each performance measure over 600 iterations were calculated.

## Results

Patient characteristics are shown in Table 1. Anonymized study data are shown in S1 File. The ALC group were significantly older than the AwLC (Control) group, although this difference disappeared when adjusted for age at diagnosis of cirrhosis. They had a higher BMI, and as expected, elevated alanine aminotransferase and aspartate transaminase levels, a higher MELD score and an elevated INR. They also had decreased serum albumin, haemoglobin, white cell count and platelet count. Six of the 25 lipid classes and subclasses were significantly associated with ALC, these included the glycosphingolipids, dihexosylceramide and trihexosylceramide, the ether linked lipids alkylphosphatidylcholine and lysoalkylphosphatidylcholine as well as phosphatidylinositol and free cholesterol (Table 2). Diacylglycerol and triacylglycerol were negatively associated with ALC but this was not significant after correction for multiple comparisons. There were 18 individual lipid species that showed a significant association with ALC after correction for multiple comparisons, including most species of di- and trihexosylceramide and a number of alkyl- and alkenylphosphatidylcholine species (S2 Table). Lipid classes and subclasses associated with bilirubin included dihexosylceramide and trihexosylceramide, alkylphosphatidylcholine phosphatidylinositol and free cholesterol, while dihexosylceramide and alkylphosphatidylcholine were associated with the MELD score (Table 3).

**Table 2:** Association of lipid classes with alcoholic liver cirrhosis (versus alcoholics without liver disease).

Lipid Class	IQR Odds Ratio^1^	p-value^2^
dihydroceramide	1.28 (0.52, 3.20)	0.64
ceramide	0.52 (0.21, 1.30)	0.29
monohexosylceramide	1.80 (0.83, 3.89)	0.28
dihexosylceramide	9.02 (2.38, 34.20)	**0.0258**
trihexosylceramide	45.48 (6.53, 316.60)	**0.0076**
G**_M3_** ganglioside	1.39 (0.66, 2.93)	0.52
sphingomyelin	2.40 (0.91, 6.32)	0.19
phosphatidylcholine	0.60 (0.23, 1.56)	0.47
alkylphosphatidylcholine	12.91 (2.21, 75.22)	**0.0398**
alkenylphosphatidylcholine	2.28 (0.95, 5.49)	0.18
lysophosphatidylcholine	0.71 (0.31, 1.62)	0.52
lysoalkylphosphatidylcholine	6.25 (1.70, 23.04)	**0.0398**
phosphatidylethanolamine	1.23 (0.62, 2.45)	0.64
alkylphosphatidylethanolamine	1.48 (0.62, 3.50)	0.52
alkenylphosphatidylethanolamine	1.01 (0.44, 2.29)	0.99
lysophosphatidylethanolamine	0.79 (0.36, 1.77)	0.64
phosphatidylinositol	2.76 (1.29, 5.90)	**0.0482**
lysophosphatidylinositol	0.52 (0.20, 1.33)	0.29
phosphatidylserine	0.90 (0.57, 1.41)	0.66
phosphatidylglycerol	0.70 (0.30, 1.61)	0.52
bis(monoacylglycero)phosphate	9.91 (1.52, 64.58)	0.07
free cholesterol	3.92 (1.50, 10.22)	**0.0398**
cholesteryl ester	0.56 (0.25, 1.22)	0.28
diacylglycerol	0.32 (0.11, 0.88)	0.09
triacylglycerol	0.33 (0.13, 0.88)	0.09

^1^ Odds ratio (95% confidence interval).

^2^ p-values are corrected for multiple comparisons using the Benjamini-Hochberg method.

**Table 3:** Association of lipid classes with bilirubin and the MELD score.

Lipid Class	Bilirubin		MELD	
	β-Coefficient^1^	p-value^2^	β-Coefficient^1^	p-value^2^
dihydroceramide	8.38 (-2.89, 19.65)	0.34	-0.21 (-2.41, 1.99)	0.92
ceramide	-5.42 (-15.84, 4.99)	0.47	-1.12 (-3.12, 0.87)	0.43
monohexosylceramide	6.79 (-2.04, 15.62)	0.34	1.02 (-0.69, 2.72)	0.41
dihexosylceramide	11.55 (4.60, 18.51)	**0.0097**	1.45 (0.04, 2.85)	0.17
trihexosylceramide	13.29 (6.14, 20.43)	**0.0037**	2.41 (1.02, 3.79)	**0.0156**
G_M3_ ganglioside	5.41 (-3.74, 14.56)	0.45	0.82 (-0.94, 2.58)	0.50
sphingomyelin	8.46 (-3.42, 20.33)	0.35	1.77 (-0.50, 4.04)	0.28
phosphatidylcholine	0.19 (-11.49, 11.87)	0.97	-1.14 (-3.36, 1.07)	0.47
alkylphosphatidylcholine	19.13 (10.06, 28.20)	**0.0014**	3.53 (1.77, 5.29)	**0.0059**
alkenylphosphatidylcholine	10.30 (0.42, 20.19)	0.13	1.54 (-0.38, 3.46)	0.28
lysophosphatidylcholine	-6.17 (-16.52, 4.19)	0.45	-0.87 (-2.86, 1.13)	0.51
lysoalkylphosphatidylcholine	12.23 (2.03, 22.43)	0.07	2.68 (0.76, 4.61)	0.05
phosphatidylethanolamine	12.65 (4.45, 20.86)	**0.0159**	1.39 (-0.27, 3.05)	0.26
alkylphosphatidylethanolamine	-3.50 (-14.41, 7.41)	0.61	-0.06 (-2.16, 2.04)	0.95
phosphatidylethanolamine	-5.05 (-15.45, 5.34)	0.48	-0.13 (-2.14, 1.87)	0.93
lysophosphatidylethanolamine	5.24 (-4.24, 14.71)	0.47	0.19 (-1.65, 2.02)	0.92
phosphatidylinositol	15.06 (7.76, 22.37)	**0.0014**	1.82 (0.30, 3.34)	0.10
lysophosphatidylinositol	1.75 (-7.97, 11.46)	0.76	-1.36 (-3.19, 0.47)	0.29
phosphatidylserine	1.23 (-4.13, 6.60)	0.71	-0.43 (-1.46, 0.59)	0.51
phosphatidylglycerol	5.38 (-5.19, 15.95)	0.47	-0.47 (-2.51, 1.57)	0.78
bis(monoacylglycero)phosphate	14.20 (8.12, 20.29)	**0.0007**	2.05 (0.79, 3.31)	**0.0191**
free cholesterol	13.38 (4.18, 22.58)	**0.0218**	2.29 (0.50, 4.08)	0.08
cholesteryl ester	-3.88 (-13.28, 5.53)	0.53	-1.15 (-2.94, 0.63)	0.38
diacylglycerol	-3.40 (-14.02, 7.22)	0.61	-1.83 (-3.81, 0.15)	0.21
triacylglycerol	-3.45 (-11.71, 4.82)	0.53	-1.45 (-2.99, 0.10)	0.21

^1^ β-coefficient (95% confidence interval).

^2^ p-values are corrected for multiple comparisons using the Benjamini-Hochberg method.

**Fig 1:**
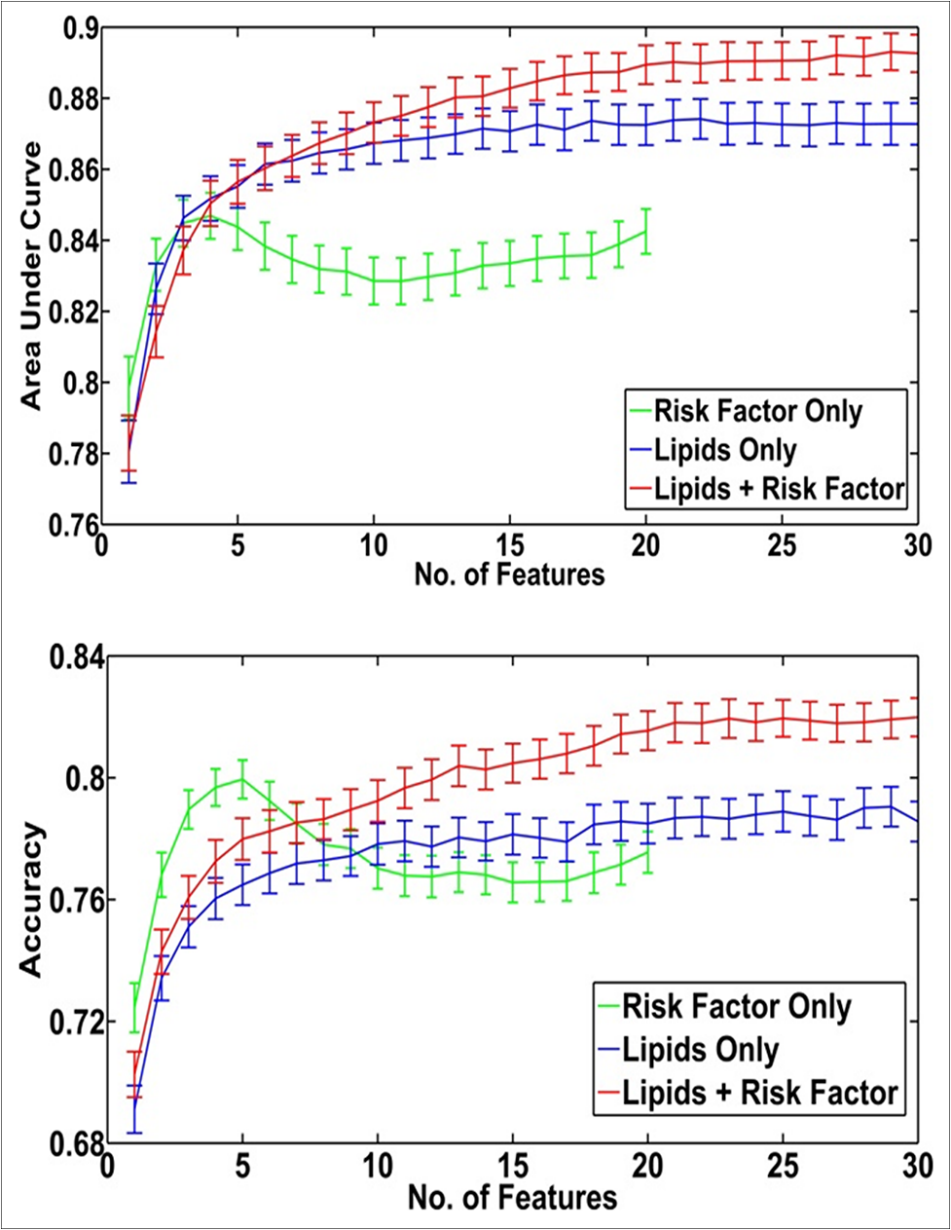
Area under the curve and accuracy for cross-validation performed on the computational models built to discriminate alcoholic liver cirrhosis. Multivariate models of increasing feature size (e.g., 1, 2, 3, 4…, 30) that included either clinical characteristics alone, lipids alone or lipids with clinical characteristics were created using the ReliefF feature selection algorithm to determine the order of feature incorporation. Models were based on a linear kernel, support vector machine based classifier within a 3-fold cross validation framework (repeated 200 times). The mean C-statistic (panel A) and percentage accuracy (panel B) from each model is plotted against the number of variables (solid lines). 95% Confidence intervals are shown by the broken lines.

Multivariate classification models were created using clinical characteristics alone, lipids alone, or a combination of lipids and clinical characteristics. The models were created using a 3 = fold cross-validation approach (repeated for 200 times). The frequency of incorporation of each feature was then calculated as the number of times the given feature was ranked within top n features of the ranking lists divided by 600; with n equal to 5 for the clinical characteristics model and 25 for lipids only and the combined (lipids and clinical characteristics) models. This serves to identify those features that are most useful within each of the models. Using only clinical characteristics resulted in an optimal model containing only four variables, with an AUC of 0.847 and an accuracy of 79.7% (Table 4, Fig 1). The most frequently utilised clinical characteristics in this model were platelets, albumin, INR and haemoglobin (Table 5). Models created with only lipids performed slightly better with an optimal AUC of 0.874 and accuracy of 78.7%, attained with a model of 22 lipid species (Table 4, Fig 1). The addition of lipids to the clinical characteristics resulted in models with the best performance with an AUC of 0.892 and accuracy of 81.8% reached with models containing 27 features (Table 4, Fig 1). Compared to the clinical characteristics model, addition of lipids in a classification model also improved net reclassification by more than 4%. The features most frequently used in these models included species of trihexosylceramide, phosphatidylcholine and lysophospholipids, in addition to platelets, INR and haemoglobin (Table 5).

**Table 4:** Classification performance of multivariate models for alcoholic liver cirrhosis^1^.

Performance Measure	Clinical Characteristics	Lipids	Lipids + Clinical Characteristics Model
No. of features required to maximise AUC	4	22	27
Accuracy	79.7 (79.0, 80.2)	78.7 (78.0, 79.4)	81.8 (81.2, 82.4)
Area Under ROC Curve	0.847 (0.840, 0.853)	0.874 (0.869, 0.88)	0.892 (0.887, 0.897)
Sensitivity	72.9 (71.7, 74.0)	72.3 (71.1, 73.6)	76.1 (75.0, 77.2)
Specificity	87.2 (86.3, 88.1)	85.8 (84.7, 86.8)	88.1 (87.2, 89.0)
Net Reclassification Index^2^	-	-	4.11%

^1^ Mean and 95% confidence intervals.

^2^ Net reclassification improvement of the combined model over the risk factor model.

**Table 5:** Frequency of feature incorporation in the alcoholic liver cirrhosis classification models.

Rank	Clinical Characteristics Only Model	Frequency %^2^	Lipids-Only Model	Frequency %^3^	Lipids and Clinical Characteristics Model	Frequency %^4^
1	Platelet	99.00	PC 32:0	98.17	PC 32:0	96.83
2	Albumin	96.17	THC 24:1	97.50	THC 24:1	94.83
3	INR	93.50	THC 20:0	94.67	THC 22:0	93.33
4	Hemoglobin	57.17	THC 22:0	94.00	THC 20:0	91.83
5	WBC	56.50	PC(O-32:0)	93.83	PC(O-32:0)	91.50
6	Bilirubin	20.50	DHC 20:0	88.67	DHC 20:0	88.83
7	BMI	13.17	PC 31:0	86.50	PC 31:0	84.83
8	Triglycerides	12.83	PC(O-34:1)	85.33	Platelet	82.50
9	Sex	10.83	PC 36:5	81.50	PC(O-34:1)	80.50
10	DBP	9.33	PC(P-34:3)	74.50	PC 36:5	73.67
11	AST/ALT^5^	7.50	LPC 20:5	74.00	DHC 22:0	72.00
12	HDL	6.50	DHC 22:0	70.83	INR	68.50
13	LDL	4.83	PC 30:0	69.00	PC(P-34:3)	68.17
14	GGT	4.00	LPE 22:6	69.00	LPC 20:5	65.50
15	Creatinine	2.50	THC 24:0	63.00	PC 30:0	62.50
16	AC^1^ (Per day)	2.33	THC 18:0	59.33	Hemoglobin	56.50
17	AC^1^ (Life time)	1.67	LPI 20:4	57.33	LPI 20:4	55.33
18	AC^1^ (Per year)	1.33	PC(O-32:1)	51.33	LPE 22:6	55.17
19	SBP	0.17	SM 33:1	45.50	THC 18:0	54.50
20	Cholesterol	0.17	LPC 20:4	41.67	THC 24:0	49.50

^1^ AC: Alcohol consumption.

^2^ Based on model containing 5 features.

^3^ Based on model containing 25 features.

^4^ Based on model containing 25 features.

^5^ Aspartate transaminase/alanine aminotransferase.

## Discussion

### Biological relevance of lipids associated with alcoholic liver cirrhosis

It is long known that alcohol consumption is associated with changes in all lipid components of plasma [21]. Alcohol enhances fatty acid synthesis, driving triglyceride synthesis, and hinders VLDL secretion leading to the accumulation of triglycerides in lipid droplets within the hepatocytes [22]. Alcohol induced fat accumulation in the hepatocytes can trigger an inflammatory response giving rise to steatohepatitis and increases the risk of progression to fibrosis and cirrhosis in some individuals, leading to serious health consequences. Identification of these individuals in the early stages of the disease would enable intervention prior to irreparable liver damage. Lipidomic technologies, now allow for the detailed analysis of individual lipid species associated with disease and their evaluation as potential biomarkers.

There are two dimensions to lipid biomarkers associated with liver disease; how far the disease has progressed (normal liver/steatosis/various stages of fibrosis/cirrhosis); and the cause of the disease (viral/NAFLD/alcohol etc.). In our study, the comparison was between individuals with ALC and AwLC, thus we are examining the more advanced stage of disease after the onset of alcoholic steatosis and establishment of cirrhosis. Our observations of several lipid classes and species showing positive associations with ALC, relative to AwLC, highlights the extensive metabolic abnormalities associated with cirrhosis. The strong positive association of both dihexosyl- and trihexosylceramide with cirrhosis contrasts with our previous observations that these glycosphingolipids are negatively associated with obesity [16] as well as with prediabetes and type 2 diabetes [15] all situations where steatosis is likely to be a contributing factor. A similar effect was observed for the alkylphosphatidylcholine and lysoalkylphosphatidylcholine that also showed positive associations with ALC (relative to AwLC) but were previously observed to have negative associations with obesity, prediabetes and type 2 diabetes [15,16]. However, while early studies showed chronic alcohol increased hepatic activity of the enzymes choline phosphotransferse and phosphatidylethanolamine methyltransferase involved in phosphatidylcholine synthesis [23] we observed an odds ratio of only 0.60 for phosphatidylcholine with ALC (non-significant) indicating that a general increase in phosphatidylcholine synthesis is not associated with progression to ALC and that this was restricted to only the ether linked phosphatidylcholine species.

We also observed negative associations of diacyl- and triacylglycerol species with ALC, with IQR odds ratios of 0.32 (95% CI 0.11–0.88) and 0.33 (95% CI 0.13–0.88) respectively, although these were non-significant after correction for multiple comparisons. Similar observations in a baboon model of alcoholic liver disease, showed that microsomal diacylglycerol acyltransferase and cytosolic phosphatidate phosphohydrolase activities increased with early stages of fatty liver which disappeared with progression of liver injury [24]. The clinical measure of total triglycerides also showed the same trend (Table 1). These multiple associations with ALC that appear to oppose those associations with steatosis and early liver disease, may relate to a decrease in free fatty acids resulting from a decrease in lipogenesis as the liver progresses to cirrhosis and the subsequent damage reduces the synthetic capacity of the liver [25] including fatty acid synthesis. These differences may also be a consequence of additional interactions of alcohol metabolites, acetaldehyde and acetate, that can form adducts thereby interfering with lipoprotein catabolism. In the present study, it should also be noted that these metabolic differences may arise as a result of some ALC patients undergoing treatment/awaiting transplant and so may not be actively drinking at the time of sample collection.

Interestingly, phosphatidylinositol, was also positively associated with cirrhosis demonstrating that not all lipid metabolic pathways are influenced in the same way. Phosphatidylinositol, is a major source of arachidonic acid, a precursor to the proinflammatory eicosanoids and so the positive association of this lipid class with both the early indicators of steatosis previously reported [15,16] and with the progression to cirrhosis, shown here, supports the role of the inflammatory state contributing to the disease progression.

Notwithstanding the opposing associations of several metabolic pathways in the ALC group, the elevated levels of dihexosylceramide may have specific effects on gene regulation and lipid metabolism leading to the increase in free cholesterol associated with cirrhosis. A recent paper by Chatterjee *et al*. examined the effect of inhibiting glycosphingolipid synthesis on atherosclerosis and arterial stiffness [26]. They observed that inhibition of glycosphingolipids synthesis resulted in a decrease in monohexosyl- and dihexosylceramide in the liver and this was associated with a decrease in serum cholesterol and triglycerides via recruitment of multiple genes/pathways of lipid metabolism. Specifically, inhibition of glycosphingolipids synthesis increased LDL receptor and SREBP2 gene expression and decreased HMGCoA reductase gene expression, suggesting inhibition of cholesterol biosynthesis and increased LDL uptake. The decreased monohexosyl- and dihexosylceramide were also associated with increased expression of genes responsible for cholesterol efflux including ABCA1, ABCG5 and ABCG8. This inhibitory effect of lactosylceramide (a dihexosylceramide) in cholesterol efflux via the ABCA1/Apolipiprotein A-1 pathway has also been demonstrated in cultured fibroblasts [27]. The decreased levels of lactosylceramide were also associated with the up regulation of the Cyp7A1 gene which encodes a 7-hydroxylase enzyme that converts cholesterol to bile acid for excretion [26]. Thus, lactosylceramide appears to influence multiple lipid metabolic pathways leading to elevated cholesterol within the liver. Elevated cholesterol within hepatic stellate cells has been shown to increase fibrosis in ACAT-1 deficient mice [28] further supporting a causal role for lipid metabolism in ALC. Dihexosylceramide then, is emerging as a key mediator of fibrosis and may play an important role in the regulation of cholesterol metabolism leading to cirrhosis. This raises the dual possibility of utilising these lipids as early indicators of cirrhosis and of targeting this metabolic pathway to prevent progression to cirrhosis. It should be noted that while the underlying dysregulation of lipid metabolism likely plays an important role in ALC, whether these metabolic changes are also present in other forms of cirrhosis remains to be determined.

### Classification of alcoholic liver cirrhosis

Despite our incomplete knowledge of the metabolic dysregulation leading to the serum lipid profiles associated with ALC we were able to evaluate these lipids as potential biomarkers to identify those individuals with established liver disease. We were clearly able to show that serum lipid species can improve on traditional clinical characteristics in classification models. The model using only clinical characteristics gave a maximum AUC and accuracy with four clinical characteristics, with platelet count, albumin, INR and haemoglobin being most often included in these models. While models developed with lipid species alone gave similar results, the combination of lipid species with the clinical characteristics resulted in models where the maximum performance was obtained with a model containing 27 features, with platelet count, INR and haemoglobin being incorporated into these models along with multiple species of lipids (Table 4). The net reclassification index (NRI) of the mixed model relative to the model using clinical characteristics only was 4.1% which while modest, highlights the potential of lipids to add to clinical characteristics. Although many lipids were associated with bilirubin, the lipids were preferentially incorporated to the model, over bilirubin, demonstrating their independent value to the model.

A recent report on a new classification model for hepatitis C virus related cirrhosis included plasma lathosterol in a multivariate model with BMI, platelet count and prothrombin index to provide a similar AUC of 0.91 (95% CI 0.82–1.0) [29] compared to our value of 0.892 (95% CI 0.887, 0.897). Interestingly the negative association of lathosterol in this study suggests a down regulation of cholesterol synthesis in hepatitis C virus related cirrhosis as opposed to the apparent increase in ALC.

### Study Limitations

Our long term goal is to identify early biomarkers of alcoholic liver disease, which could allow intervention before development of cirrhosis. We compared lipid profiles from AwLC patients (controls) to those with advanced alcoholic cirrhosis to determine lipid biomarkers for the identification of ALC within the high risk alcohol consuming group to provide the clearest lipid signature characteristic of disease progression. The current cohort is of moderate size and consists of patients with advanced ALC compared to AwLC. The large clinical difference between these groups may have resulted in inflated odds ratios because of spectrum bias [30], for some lipid species. We believe the inclusion of early stage alcoholic liver disease patients in future studies and the analysis of prospective cohorts will further strengthen our results. Validation of lipid species identified in this study in patients with early stages of ALC is warranted as are studies to assess other forms of liver disease with different etiologies. For classification modelling, we have employed a 3-fold cross-validation framework repeated 200 times to resample the data to overcome the limited sample size available. We have assessed the performance of the classification models by computing a number of performance measures and their confidence intervals. The gain in AUC and NRI values demonstrate the value of lipid species as features in the classification model over clinical characteristics. We were however, unable to obtain an independent validation cohort to confirm the performance of the classification model.

## Supporting Information

S1 File Anonymized Data—ALC Study. (XLSX)

S1 Table Inclusion / Exclusion selection criteria for cases and controls. (DOCX)

S2 Table Association of lipid species with alcoholic liver cirrhosis. (DOCX)
