# Supplementary material for: Circulating Lipids Are Associated with Alcoholic Liver Cirrhosis and Represent Potential Biomarkers for Risk Assessment
Source: PLoS One. 2015 Jun 24;10(6):e0130346. doi: 10.1371/journal.pone.0130346 (PMC4479371; doi:10.1371/journal.pone.0130346)
Supplement: S1 Table — (DOCX) [file pone.0130346.s002.docx]

**Supporting Information**

**S1 Table. Inclusion / Exclusion selection criteria for cases and controls**

| **CASES: Heavy drinkers with alcoholic liver cirrhosis** | | |
| --- | --- | --- |
| **Inclusion** | **Exclusion^1^** | |
| 1. Average daily ethanol consumption of > 50 grams for women and > 80 grams for men for more than 10 years. Judgment regarding daily and yearly alcohol use will be made by the site investigator 2. Cirrhosis based on one or more of the following clinical, histological or FibroScan criteria.    1. Clinical cirrhosis documentation in the medical record of one or more of the following:  - clinically detectable ascites (confirmed by imaging or by paracentesis), - spontaneous hepatic encephalopathy (grade 2 or higher), - moderate or large esophageal varices on upper endoscopy, or   1. Histological cirrhosis: Metavir fibrosis stage F4 or Ishak fibrosis stage 5 or 6   2. Fibroscan: Adequately performed FibroScan with stiffness for F4 cut off is ≥22 kPa if AST <100 IU/L within 2 weeks of FibroScan, OR   If AST between 100-200 IU/L within 2 weeks of FibroScan, then F4 cut off is ≥30 kPa. Adequately performed FibroScan is defined as at least 10 adequate measurements and IQR <30% in a subject who has fasted for ≥2 hours prior to FibroScan examination. | 1. Liver disease other than alcoholic liver disease. The following diseases must excluded using the noted test:    1. Hepatitis C: negative hepatitis C antibody. Can include patients with HCV antibody but undetectable hepatitis C viral load, if there is no history of antiviral therapy for hepatitis C.    2. Hepatitis B: negative HBsAg.    3. Hemochromatosis: subjects with a transferrin saturation >45% or with significant iron (i.e., > 2+ iron) on liver biopsy must be tested for genetic hemochromatosis. Subjects homozygous for C282Y or with C282Y/H63D compound heterozygous results are excluded.    4. Autoimmune liver disease: undetectable or low ANA titer. Other tests (e.g., smooth muscle antibody) must not be present at levels which would suggest autoimmune liver disease.    5. Wilson disease: normal serum ceruloplasmin level.   Patients with a decreased ceruloplasmin level must have a liver biopsy which excludes the presence of excess copper binding protein   1. Liver transplantation for liver disease other than ALC 2. Known or suspected HIV infection. (NOTE: Patients will not be tested for HIV) | |
| **CONTROLS: Heavy drinkers without liver cirrhosis** | | |
| **Inclusion** | **Exclusion*** | |
| 1. Average daily consumption of greater than 50 grams of ethanol/day for women and greater than 80 grams/day for males for more than 10 years. Judgment regarding daily and yearly alcohol use will be made by the site investigator  2. Abstinence from heavy, daily alcohol use (see above) of less than 60 days  3. Lack of significant liver disease as documented by one of the following:  a. Blood tests while actively drinking or within 7 days of stopping the most recent episode of heavy alcohol use which demonstrate normal results for AST, ALT, total bilirubin, albumin and INR, or  b. Fibroscan: Adequately performed FibroScan with stiffness ≤6.0 kPa (even with an elevated AST). Adequately performed FibroScan is defined as at least 10 adequate measurements and IQR <30% in a subject who has fasted for ≥2 hours prior to FibroScan examination. | 1. Any exclusion listed above for CASES (items 1 – 3)  2. Prior hepatic decompensation. This includes a history of jaundice, ascites, variceal bleeding, upper gastrointestinal bleeding of uncertain etiology, or blood tests which suggest impaired liver function or acute/chronic alcoholic liver injury  3. Liver biopsy is not required. However, if liver biopsy has been performed, presence of the following histological findings would exclude the patient:   - 1. F1, F2, F3 or F4 fibrosis   2. Pericellular (i.e., intrasinusoidal) fibrosis   3. Perivenular fibrosis   4. Alcoholic hepatitis | |
| **Matching CASES and CONTROLS** | | |
| 1. Gender: Male/Female  2. Age: Controls within-3 to +5 years of Case at the time of first diagnosis of cirrhosis (either clinical or histological) | | 3. Race/Ethnicity Matched according to individual country criteria  4. Country: Preferably from same hospital |

*^1^* Patients with risk factors for non-alcoholic fatty liver (e.g., overweight, insulin resistance, high triglycerides) can be enrolled into this study provided they have consumed sufficient ethanol.
