# Supplementary material for: Circulating Lipids Are Associated with Alcoholic Liver Cirrhosis and Represent Potential Biomarkers for Risk Assessment
Source: PLoS One. 2015 Jun 24;10(6):e0130346. doi: 10.1371/journal.pone.0130346 (PMC4479371; doi:10.1371/journal.pone.0130346)
Supplement: S2 Table — (DOCX) [file pone.0130346.s003.docx]

**Supporting Information**

**S3 Table. Association of lipid species with alcoholic liver cirrhosis**

| No. | Lipid Species^1^ | IQR Odds Ratio^2^ | Uncorrected  p-value^3^ | Corrected p-value^4^ |
| --- | --- | --- | --- | --- |
| 1 | Cer d18:0/16:0 | 2.02 (0.98, 4.16) | 0.06 | 0.15 |
| 2 | Cer d18:0/18:0 | 0.82 (0.39, 1.74) | 0.61 | 0.71 |
| 3 | Cer d18:0/20:0 | 1.02 (0.44, 2.36) | 0.96 | 0.97 |
| 4 | Cer d18:0/22:0 | 1.33 (0.61, 2.91) | 0.48 | 0.59 |
| 5 | Cer d18:0/24:0 | 1.04 (0.46, 2.34) | 0.92 | 0.95 |
| 6 | Cer d18:0/24:1 | 1.33 (0.66, 2.69) | 0.43 | 0.55 |
| 7 | Cer d18:1/16:0 | 6.31 (1.34, 29.74) | **0.0235** | 0.10 |
| 8 | Cer d18:1/18:0 | 0.89 (0.52, 1.52) | 0.68 | 0.75 |
| 9 | Cer d18:1/20:0 | 0.70 (0.32, 1.51) | 0.36 | 0.49 |
| 10 | Cer d18:1/22:0 | 0.62 (0.28, 1.37) | 0.24 | 0.37 |
| 11 | Cer d18:1/24:0 | 0.44 (0.19, 1.00) | 0.06 | 0.14 |
| 12 | Cer d18:1/24:1 | 0.74 (0.38, 1.44) | 0.38 | 0.51 |
| 13 | HexCer d18:1/16:0 | 1.94 (0.80, 4.72) | 0.15 | 0.27 |
| 14 | HexCer d18:1/18:0 | 1.17 (0.69, 1.99) | 0.56 | 0.67 |
| 15 | HexCer d18:1/20:0 | 1.85 (0.88, 3.88) | 0.11 | 0.22 |
| 16 | HexCer d18:1/22:0 | 2.62 (1.03, 6.65) | **0.0476** | 0.13 |
| 17 | HexCer d18:1/24:0 | 1.30 (0.60, 2.78) | 0.51 | 0.62 |
| 18 | HexCer d18:1/24:1 | 1.98 (0.82, 4.80) | 0.13 | 0.25 |
| 19 | Hex2Cer d18:1/16:0 | 3.48 (1.44, 8.39) | **0.0076** | 0.07 |
| 20 | Hex2Cer d18:1/18:0 | 10.48 (2.92, 37.62) | **0.0007** | **0.0265** |
| 21 | Hex2Cer d18:1/20:0 | 35.51 (5.51, 228.69) | **0.0004** | **0.0265** |
| 22 | Hex2Cer d18:1/22:0 | 25.85 (4.97, 134.48) | **0.0003** | **0.0265** |
| 23 | Hex2Cer d18:1/24:0 | 5.44 (1.90, 15.59) | **0.0026** | **0.0495** |
| 24 | Hex2Cer d18:1/24:1 | 16.22 (2.90, 90.79) | **0.0025** | **0.0495** |
| 25 | Hex3Cer d18:1/16:0 | 18.81 (3.31, 106.84) | **0.0016** | **0.0394** |
| 26 | Hex3Cer d18:1/18:0 | 18.77 (3.68, 95.71) | **0.0009** | **0.0265** |
| 27 | Hex3Cer d18:1/20:0 | 78.29 (9.82, 624.43) | **0.0001** | **0.0218** |
| 28 | Hex3Cer d18:1/22:0 | 50.27 (8.38, 301.67) | **7.42E-05** | **0.0218** |
| 29 | Hex3Cer d18:1/24:0 | 13.08 (3.17, 53.90) | **0.0008** | **0.0265** |
| 30 | Hex3Cer d18:1/24:1 | 25.70 (4.79, 137.93) | **0.0004** | **0.0265** |
| 31 | GM3 d18:1/16:0 | 4.53 (1.40, 14.60) | **0.0144** | 0.08 |
| 32 | GM3 d18:1/18:0 | 0.57 (0.21, 1.54) | 0.27 | 0.40 |
| 33 | GM3 d18:1/20:0 | 1.15 (0.51, 2.63) | 0.74 | 0.80 |
| 34 | GM3 d18:1/22:0 | 0.90 (0.37, 2.19) | 0.81 | 0.86 |
| 35 | GM3 d18:1/24:0 | 0.61 (0.24, 1.53) | 0.30 | 0.43 |
| 36 | GM3 d18:1/24:1 | 1.31 (0.68, 2.56) | 0.42 | 0.55 |
| 37 | SM d31:1 | 5.69 (1.75, 18.47) | **0.0054** | 0.06 |
| 38 | SM d32:0 | 3.71 (1.37, 10.03) | **0.0124** | 0.08 |
| 39 | SM d32:1 | 5.56 (1.77, 17.43) | **0.0048** | 0.06 |
| 40 | SM d32:2 | 2.95 (1.02, 8.51) | 0.05 | 0.13 |
| 41 | SM d33:1 | 7.83 (2.17, 28.21) | **0.0027** | **0.0495** |
| 42 | SM d34:0 | 2.45 (1.22, 4.92) | **0.0143** | 0.08 |
| 43 | SM d34:1 | 3.38 (1.31, 8.67) | **0.0143** | 0.08 |
| 44 | SM d34:2 | 1.80 (0.80, 4.05) | 0.16 | 0.28 |
| 45 | SM d34:3 | 1.88 (0.75, 4.71) | 0.18 | 0.30 |
| 46 | SM d35:1 | 2.14 (0.88, 5.23) | 0.10 | 0.21 |
| 47 | SM d35:2 | 1.61 (0.74, 3.52) | 0.24 | 0.36 |
| 48 | SM d36:1 | 0.56 (0.23, 1.36) | 0.20 | 0.33 |
| 49 | SM d36:2 | 0.67 (0.31, 1.45) | 0.31 | 0.44 |
| 50 | SM d36:3 | 1.07 (0.52, 2.22) | 0.86 | 0.90 |
| 51 | SM d37:2 | 1.56 (0.70, 3.48) | 0.28 | 0.41 |
| 52 | SM d38:1 | 0.61 (0.27, 1.41) | 0.25 | 0.38 |
| 53 | SM d38:2 | 0.73 (0.30, 1.74) | 0.48 | 0.59 |
| 54 | SM d39:1 | 2.61 (0.99, 6.88) | 0.06 | 0.14 |
| 55 | SM d41:1 | 1.09 (0.45, 2.64) | 0.85 | 0.89 |
| 56 | SM d41:2 | 1.61 (0.64, 4.05) | 0.32 | 0.44 |
| 57 | SM d42:1 | 0.52 (0.22, 1.26) | 0.15 | 0.27 |
| 58 | PC 28:0 | 2.64 (1.08, 6.47) | **0.0384** | 0.11 |
| 59 | PC 29:0 | 2.71 (1.07, 6.87) | **0.0396** | 0.12 |
| 60 | PC 30:0 | 10.78 (2.23, 52.13) | **0.0046** | 0.06 |
| 61 | PC 31:0 | 6.71 (1.48, 30.39) | **0.0166** | 0.09 |
| 62 | PC 31:1 | 4.04 (1.29, 12.67) | **0.0201** | 0.09 |
| 63 | PC 32:0 | 20.26 (3.97, 103.50) | **0.0007** | **0.0265** |
| 64 | PC 32:1 | 1.21 (0.58, 2.51) | 0.61 | 0.71 |
| 65 | PC 32:2 | 0.82 (0.34, 1.98) | 0.66 | 0.74 |
| 66 | PC 32:3 | 0.82 (0.38, 1.79) | 0.62 | 0.71 |
| 67 | PC 33:0 | 6.73 (1.90, 23.89) | **0.0047** | 0.06 |
| 68 | PC 33:1 | 1.87 (0.87, 3.99) | 0.11 | 0.23 |
| 69 | PC 33:2 | 1.11 (0.51, 2.40) | 0.80 | 0.85 |
| 70 | PC 33:3 | 1.16 (0.54, 2.51) | 0.70 | 0.78 |
| 71 | PC 34:0 | 4.92 (1.69, 14.36) | **0.0051** | 0.06 |
| 72 | PC 34:1 | 0.77 (0.35, 1.71) | 0.53 | 0.64 |
| 73 | PC 34:2 | 0.64 (0.28, 1.49) | 0.31 | 0.44 |
| 74 | PC 34:3 | 0.88 (0.38, 2.06) | 0.77 | 0.83 |
| 75 | PC 34:4 | 0.60 (0.26, 1.38) | 0.23 | 0.36 |
| 76 | PC 34:5 | 0.65 (0.38, 1.10) | 0.12 | 0.23 |
| 77 | PC 35:0 | 3.17 (1.17, 8.62) | **0.0275** | 0.10 |
| 78 | PC 35:1 | 1.24 (0.73, 2.11) | 0.43 | 0.55 |
| 79 | PC 35:2 | 1.09 (0.49, 2.43) | 0.83 | 0.88 |
| 80 | PC 35:3 | 1.04 (0.47, 2.28) | 0.93 | 0.95 |
| 81 | PC 35:4 | 0.62 (0.29, 1.35) | 0.23 | 0.36 |
| 82 | PC 35:5 | 0.39 (0.15, 1.03) | 0.06 | 0.15 |
| 83 | PC 36:0 | 2.31 (0.98, 5.45) | 0.06 | 0.15 |
| 84 | PC 36:1 | 2.01 (0.87, 4.67) | 0.11 | 0.23 |
| 85 | PC 36:2 | 1.28 (0.64, 2.58) | 0.49 | 0.61 |
| 86 | PC 36:3 | 0.91 (0.38, 2.17) | 0.83 | 0.88 |
| 87 | PC 36:4b | 0.29 (0.10, 0.80) | **0.0201** | 0.09 |
| 88 | PC 36:5 | 0.34 (0.13, 0.92) | **0.0371** | 0.11 |
| 89 | PC 36:6 | 0.48 (0.21, 1.07) | 0.08 | 0.17 |
| 90 | PC 37:4 | 0.93 (0.42, 2.10) | 0.87 | 0.91 |
| 91 | PC 37:5 | 0.64 (0.31, 1.29) | 0.22 | 0.34 |
| 92 | PC 37:6 | 0.49 (0.23, 1.06) | 0.08 | 0.17 |
| 93 | PC 38:2 | 1.01 (0.41, 2.49) | 0.98 | 0.99 |
| 94 | PC 38:3 | 0.74 (0.31, 1.77) | 0.50 | 0.61 |
| 95 | PC 38:4 | 0.65 (0.33, 1.29) | 0.22 | 0.35 |
| 96 | PC 38:5 | 0.37 (0.14, 0.97) | **0.0481** | 0.13 |
| 97 | PC 38:6a | 0.69 (0.32, 1.49) | 0.35 | 0.48 |
| 98 | PC 38:6b | 0.19 (0.06, 0.63) | **0.0082** | 0.07 |
| 99 | PC 38:7 | 0.39 (0.15, 0.98) | 0.05 | 0.13 |
| 100 | PC 39:5 | 0.85 (0.47, 1.52) | 0.58 | 0.69 |
| 101 | PC 39:6 | 0.74 (0.39, 1.42) | 0.37 | 0.50 |
| 102 | PC 39:7 | 0.70 (0.39, 1.27) | 0.25 | 0.37 |
| 103 | PC 40:4 | 1.22 (0.57, 2.62) | 0.61 | 0.71 |
| 104 | PC 40:5 | 0.65 (0.31, 1.33) | 0.24 | 0.37 |
| 105 | PC 40:6 | 0.39 (0.16, 0.92) | **0.0355** | 0.11 |
| 106 | PC 40:7 | 0.52 (0.22, 1.23) | 0.14 | 0.26 |
| 107 | PC 40:8 | 0.45 (0.15, 1.35) | 0.16 | 0.28 |
| 108 | PC O-32:0 | 30.36 (4.66, 197.65) | **0.0007** | **0.0265** |
| 109 | PC O-32:1 | 6.55 (1.82, 23.56) | **0.0057** | 0.06 |
| 110 | PC O-32:2 | 6.40 (1.95, 20.98) | **0.0034** | 0.06 |
| 111 | PC O-34:1 | 12.65 (2.61, 61.27) | **0.0026** | **0.0495** |
| 112 | PC O-34:2 | 8.93 (1.75, 45.63) | **0.0111** | 0.08 |
| 113 | PC O-34:3 | 4.03 (1.28, 12.71) | **0.0207** | 0.09 |
| 114 | PC O-34:4 | 2.87 (1.04, 7.96) | **0.0473** | 0.13 |
| 115 | PC O-35:4 | 2.14 (0.89, 5.11) | 0.09 | 0.20 |
| 116 | PC O-36:0 | 5.22 (1.72, 15.90) | **0.0052** | 0.06 |
| 117 | PC O-36:1 | 10.49 (2.70, 40.84) | **0.0013** | **0.0359** |
| 118 | PC O-36:2 | 7.90 (1.84, 34.02) | **0.0075** | 0.07 |
| 119 | PC O-36:3 | 11.09 (1.98, 62.10) | **0.0083** | 0.07 |
| 120 | PC O-36:4 | 4.78 (1.49, 15.35) | **0.0111** | 0.08 |
| 121 | PC O-36:5 | 1.17 (0.54, 2.52) | 0.69 | 0.77 |
| 122 | PC O-38:4 | 8.86 (2.49, 31.57) | **0.0014** | **0.0359** |
| 123 | PC O-38:5 | 10.43 (1.25, 87.24) | **0.0349** | 0.11 |
| 124 | PC O-40:5 | 7.42 (2.03, 27.16) | **0.0037** | 0.06 |
| 125 | PC O-40:6 | 3.01 (1.11, 8.14) | **0.0346** | 0.11 |
| 126 | PC O-40:7 | 2.59 (0.88, 7.60) | 0.09 | 0.19 |
| 127 | PC P-32:0 | 5.27 (1.80, 15.39) | **0.0036** | 0.06 |
| 128 | PC P-32:1 | 5.06 (1.59, 16.08) | **0.0080** | 0.07 |
| 129 | PC P-34:1 | 4.23 (1.30, 13.84) | **0.0203** | 0.09 |
| 130 | PC P-34:2 | 2.64 (0.99, 7.01) | 0.06 | 0.14 |
| 131 | PC P-34:3 | 21.09 (3.87, 115.12) | **0.0009** | **0.0265** |
| 132 | PC P-36:2 | 3.31 (1.27, 8.63) | **0.0174** | 0.09 |
| 133 | PC P-36:4 | 1.27 (0.60, 2.69) | 0.53 | 0.64 |
| 134 | PC P-36:5 | 0.73 (0.35, 1.53) | 0.41 | 0.54 |
| 135 | PC P-38:4 | 1.73 (0.73, 4.11) | 0.22 | 0.35 |
| 136 | PC P-38:5 | 1.32 (0.59, 2.94) | 0.51 | 0.62 |
| 137 | PC P-38:6 | 0.68 (0.26, 1.79) | 0.44 | 0.56 |
| 138 | PC P-40:6 | 1.01 (0.44, 2.31) | 0.98 | 0.99 |
| 139 | LPC 14:0 | 0.98 (0.43, 2.22) | 0.96 | 0.97 |
| 140 | LPC 15:0 | 0.99 (0.52, 1.92) | 0.99 | 0.99 |
| 141 | LPC 16:0 | 0.59 (0.23, 1.52) | 0.28 | 0.41 |
| 142 | LPC 16:1 | 0.98 (0.48, 1.99) | 0.96 | 0.97 |
| 143 | LPC 17:0 | 0.99 (0.51, 1.92) | 0.99 | 0.99 |
| 144 | LPC 17:1 | 1.14 (0.58, 2.26) | 0.71 | 0.78 |
| 145 | LPC 18:0 | 1.21 (0.53, 2.80) | 0.65 | 0.74 |
| 146 | LPC 18:1 | 1.45 (0.67, 3.13) | 0.35 | 0.48 |
| 147 | LPC 18:2 | 0.57 (0.24, 1.34) | 0.20 | 0.33 |
| 148 | LPC 18:3 | 0.67 (0.32, 1.42) | 0.30 | 0.43 |
| 149 | LPC 20:0 | 2.61 (1.04, 6.53) | **0.0448** | 0.13 |
| 150 | LPC 20:1 | 4.07 (1.57, 10.52) | **0.0054** | 0.06 |
| 151 | LPC 20:2 | 1.90 (0.84, 4.31) | 0.13 | 0.24 |
| 152 | LPC 20:3 | 0.56 (0.25, 1.22) | 0.15 | 0.27 |
| 153 | LPC 20:4 | 0.55 (0.22, 1.38) | 0.21 | 0.33 |
| 154 | LPC 20:5 | 0.47 (0.22, 1.03) | 0.07 | 0.16 |
| 155 | LPC 22:0 | 3.28 (1.28, 8.41) | **0.0166** | 0.09 |
| 156 | LPC 22:1 | 4.00 (1.28, 12.51) | **0.0207** | 0.09 |
| 157 | LPC 22:5 | 0.71 (0.35, 1.43) | 0.34 | 0.47 |
| 158 | LPC 22:6 | 0.38 (0.16, 0.90) | **0.0327** | 0.11 |
| 159 | LPC 24:0 | 4.05 (1.60, 10.25) | **0.0047** | 0.06 |
| 160 | LPC 26:0 | 2.08 (0.94, 4.61) | 0.08 | 0.17 |
| 161 | LPC O-16:0 | 4.52 (1.46, 13.92) | **0.0112** | 0.08 |
| 162 | LPC O-18:0 | 4.08 (1.40, 11.89) | **0.0125** | 0.08 |
| 163 | LPC O-18:1 | 11.69 (1.35, 101.16) | **0.0296** | 0.11 |
| 164 | LPC O-20:0 | 2.27 (1.12, 4.61) | **0.0272** | 0.10 |
| 165 | LPC O-22:0 | 2.72 (1.30, 5.67) | **0.0100** | 0.08 |
| 166 | LPC O-22:1 | 2.32 (0.93, 5.80) | 0.08 | 0.17 |
| 167 | LPC O-24:0 | 2.81 (1.26, 6.26) | **0.0142** | 0.08 |
| 168 | LPC O-24:1 | 1.99 (0.80, 4.96) | 0.14 | 0.26 |
| 169 | LPC O-24:2 | 2.81 (1.16, 6.83) | **0.0264** | 0.10 |
| 170 | PE 32:0 | 1.05 (0.48, 2.29) | 0.91 | 0.94 |
| 171 | PE 32:1 | 1.25 (0.62, 2.56) | 0.54 | 0.64 |
| 172 | PE 34:1 | 1.48 (0.65, 3.38) | 0.35 | 0.48 |
| 173 | PE 34:2 | 1.69 (0.81, 3.53) | 0.17 | 0.29 |
| 174 | PE 34:3 | 1.56 (0.79, 3.07) | 0.20 | 0.33 |
| 175 | PE 35:1 | 1.58 (0.78, 3.20) | 0.21 | 0.34 |
| 176 | PE 35:2 | 1.89 (0.85, 4.20) | 0.12 | 0.24 |
| 177 | PE 36:1 | 1.85 (0.75, 4.59) | 0.19 | 0.31 |
| 178 | PE 36:2 | 1.42 (0.78, 2.55) | 0.25 | 0.38 |
| 179 | PE 36:3 | 1.67 (0.80, 3.49) | 0.18 | 0.30 |
| 180 | PE 36:4 | 0.96 (0.52, 1.77) | 0.90 | 0.93 |
| 181 | PE 36:5 | 0.94 (0.53, 1.67) | 0.82 | 0.87 |
| 182 | PE 38:3 | 0.84 (0.39, 1.79) | 0.65 | 0.74 |
| 183 | PE 38:4 | 0.62 (0.27, 1.43) | 0.27 | 0.39 |
| 184 | PE 38:5 | 0.85 (0.46, 1.56) | 0.59 | 0.70 |
| 185 | PE 38:6 | 0.68 (0.32, 1.43) | 0.31 | 0.44 |
| 186 | PE 40:5 | 0.84 (0.41, 1.70) | 0.62 | 0.71 |
| 187 | PE 40:6 | 0.63 (0.31, 1.26) | 0.20 | 0.32 |
| 188 | PE 40:7 | 0.84 (0.50, 1.39) | 0.50 | 0.61 |
| 189 | PE O-34:1 | 3.20 (1.25, 8.19) | **0.0185** | 0.09 |
| 190 | PE O-34:2 | 3.62 (1.26, 10.41) | **0.0205** | 0.09 |
| 191 | PE O-36:2 | 2.76 (1.22, 6.21) | **0.0175** | 0.09 |
| 192 | PE O-36:3 | 2.15 (0.83, 5.53) | 0.12 | 0.23 |
| 193 | PE O-36:4 | 1.11 (0.52, 2.41) | 0.78 | 0.84 |
| 194 | PE O-36:5 | 0.86 (0.48, 1.53) | 0.60 | 0.70 |
| 195 | PE O-36:6 | 0.64 (0.27, 1.50) | 0.31 | 0.44 |
| 196 | PE O-38:4 | 1.21 (0.61, 2.43) | 0.59 | 0.69 |
| 197 | PE O-38:5 | 1.15 (0.51, 2.62) | 0.73 | 0.80 |
| 198 | PE O-40:5 | 2.36 (0.97, 5.71) | 0.06 | 0.15 |
| 199 | PE O-40:6 | 1.02 (0.64, 1.62) | 0.95 | 0.97 |
| 200 | PE O-40:7 | 0.62 (0.28, 1.37) | 0.24 | 0.36 |
| 201 | PE P-34:1 | 3.74 (1.29, 10.83) | **0.0181** | 0.09 |
| 202 | PE P-34:2 | 2.50 (1.11, 5.62) | **0.0306** | 0.11 |
| 203 | PE P-36:1 | 2.02 (1.07, 3.80) | **0.0341** | 0.11 |
| 204 | PE P-36:2 | 2.72 (1.30, 5.70) | **0.0103** | 0.08 |
| 205 | PE P-36:4 | 0.86 (0.48, 1.53) | 0.60 | 0.70 |
| 206 | PE P-38:4 | 0.85 (0.42, 1.73) | 0.66 | 0.74 |
| 207 | PE P-38:5 | 0.53 (0.22, 1.30) | 0.17 | 0.29 |
| 208 | PE P-38:6 | 0.63 (0.31, 1.27) | 0.20 | 0.33 |
| 209 | PE P-40:5 | 0.86 (0.49, 1.50) | 0.59 | 0.70 |
| 210 | PE P-40:6 | 0.68 (0.28, 1.65) | 0.40 | 0.53 |
| 211 | LPE 16:0 | 0.73 (0.33, 1.63) | 0.45 | 0.57 |
| 212 | LPE 18:0 | 1.12 (0.49, 2.55) | 0.79 | 0.85 |
| 213 | LPE 18:1 | 1.25 (0.74, 2.11) | 0.41 | 0.54 |
| 214 | LPE 18:2 | 0.86 (0.44, 1.68) | 0.65 | 0.74 |
| 215 | LPE 20:4 | 0.34 (0.14, 0.83) | **0.0210** | 0.09 |
| 216 | LPE 22:6 | 0.31 (0.11, 0.86) | **0.0286** | 0.10 |
| 217 | PI 32:0 | 3.23 (1.26, 8.26) | **0.0178** | 0.09 |
| 218 | PI 32:1 | 1.57 (0.86, 2.86) | 0.15 | 0.27 |
| 219 | PI 34:0 | 2.45 (1.22, 4.92) | **0.0146** | 0.08 |
| 220 | PI 34:1 | 2.68 (1.34, 5.36) | **0.0071** | 0.07 |
| 221 | PI 36:1 | 4.58 (1.74, 12.07) | **0.0032** | 0.06 |
| 222 | PI 36:2 | 3.96 (1.45, 10.79) | **0.0094** | 0.08 |
| 223 | PI 36:3 | 5.06 (1.59, 16.09) | **0.0081** | 0.07 |
| 224 | PI 36:4 | 1.69 (0.77, 3.73) | 0.20 | 0.33 |
| 225 | PI 38:2 | 2.96 (1.26, 6.99) | **0.0162** | 0.09 |
| 226 | PI 38:3 | 1.44 (0.60, 3.45) | 0.41 | 0.54 |
| 227 | PI 38:4 | 0.67 (0.26, 1.73) | 0.41 | 0.54 |
| 228 | PI 38:5 | 4.69 (1.52, 14.48) | **0.0096** | 0.08 |
| 229 | PI 38:6 | 1.44 (0.73, 2.88) | 0.30 | 0.43 |
| 230 | PI 40:4 | 1.52 (0.62, 3.75) | 0.37 | 0.50 |
| 231 | PI 40:5 | 0.72 (0.31, 1.65) | 0.44 | 0.56 |
| 232 | PI 40:6 | 0.53 (0.21, 1.33) | 0.18 | 0.30 |
| 233 | LPI 18:0 | 0.43 (0.15, 1.24) | 0.12 | 0.24 |
| 234 | LPI 18:1 | 1.07 (0.58, 1.97) | 0.82 | 0.87 |
| 235 | LPI 18:2 | 0.44 (0.16, 1.22) | 0.12 | 0.23 |
| 236 | LPI 20:4 | 0.26 (0.08, 0.85) | **0.0308** | 0.11 |
| 237 | PS 36:1 | 0.70 (0.35, 1.38) | 0.30 | 0.44 |
| 238 | PS 36:2 | 1.22 (0.70, 2.13) | 0.48 | 0.59 |
| 239 | PS 38:3 | 1.12 (0.59, 2.12) | 0.73 | 0.80 |
| 240 | PS 38:4 | 0.91 (0.57, 1.48) | 0.71 | 0.78 |
| 241 | PS 40:5 | 1.04 (0.61, 1.77) | 0.89 | 0.93 |
| 242 | PS 40:6 | 1.00 (0.64, 1.57) | 0.99 | 0.99 |
| 243 | PG 34:1 | 1.58 (0.62, 4.02) | 0.34 | 0.47 |
| 244 | PG 36:1 | 0.76 (0.38, 1.52) | 0.44 | 0.56 |
| 245 | PG 36:2 | 0.51 (0.20, 1.32) | 0.17 | 0.29 |
| 246 | BMP 36:2 | 9.91 (1.52, 64.58) | **0.0199** | 0.09 |
| 247 | ST 27:1/OH | 3.92 (1.50, 10.22) | **0.0072** | 0.07 |
| 248 | CE 14:0 | 1.16 (0.77, 1.75) | 0.49 | 0.61 |
| 249 | CE 15:0 | 1.65 (0.81, 3.33) | 0.17 | 0.29 |
| 250 | CE 16:0 | 0.70 (0.38, 1.27) | 0.25 | 0.37 |
| 251 | CE 16:1 | 0.71 (0.33, 1.50) | 0.37 | 0.50 |
| 252 | CE 16:2 | 1.36 (0.61, 3.06) | 0.46 | 0.58 |
| 253 | CE 17:0 | 1.23 (0.60, 2.52) | 0.58 | 0.69 |
| 254 | CE 17:1 | 0.75 (0.34, 1.64) | 0.47 | 0.59 |
| 255 | CE 18:0 | 1.16 (0.51, 2.64) | 0.72 | 0.79 |
| 256 | CE 18:1 | 0.46 (0.18, 1.18) | 0.11 | 0.23 |
| 257 | CE 18:2 | 0.50 (0.21, 1.18) | 0.12 | 0.23 |
| 258 | CE 18:3 | 0.66 (0.30, 1.47) | 0.32 | 0.44 |
| 259 | CE 20:1 | 4.34 (1.59, 11.85) | **0.0059** | 0.06 |
| 260 | CE 20:3 | 0.44 (0.15, 1.25) | 0.13 | 0.24 |
| 261 | CE 20:4 | 0.46 (0.20, 1.06) | 0.07 | 0.17 |
| 262 | CE 20:5 | 0.41 (0.16, 1.05) | 0.07 | 0.16 |
| 263 | CE 22:0 | 1.59 (0.83, 3.07) | 0.17 | 0.29 |
| 264 | CE 22:1 | 3.72 (1.42, 9.76) | **0.0099** | 0.08 |
| 265 | CE 22:4 | 1.36 (0.72, 2.56) | 0.34 | 0.48 |
| 266 | CE 22:5 | 0.55 (0.26, 1.16) | 0.12 | 0.23 |
| 267 | CE 22:6 | 0.41 (0.18, 0.93) | **0.0377** | 0.11 |
| 268 | CE 24:0 | 1.57 (0.77, 3.21) | 0.22 | 0.35 |
| 269 | CE 24:1 | 2.93 (1.32, 6.50) | **0.0108** | 0.08 |
| 270 | CE 24:5 | 2.54 (1.18, 5.45) | **0.0204** | 0.09 |
| 271 | CE 24:6 | 1.10 (0.68, 1.77) | 0.71 | 0.78 |
| 272 | DG 14:0_18:1 | 0.48 (0.24, 0.95) | **0.0400** | 0.12 |
| 273 | DG 14:0_18:2 | 0.49 (0.26, 0.95) | **0.0383** | 0.11 |
| 274 | DG 16:0_18:1 | 0.35 (0.13, 0.91) | **0.0352** | 0.11 |
| 275 | DG 16:0_18:2 | 0.42 (0.19, 0.95) | **0.0418** | 0.12 |
| 276 | DG 16:0_20:0 | 1.38 (0.62, 3.09) | 0.43 | 0.56 |
| 277 | DG 16:0_20:3 | 0.85 (0.41, 1.79) | 0.67 | 0.75 |
| 278 | DG 16:0_20:4 | 0.62 (0.35, 1.08) | 0.10 | 0.21 |
| 279 | DG 16:0_22:5 | 0.53 (0.27, 1.06) | 0.08 | 0.17 |
| 280 | DG 16:0_22:6 | 0.35 (0.14, 0.87) | **0.0271** | 0.10 |
| 281 | DG 16:1_18:0 | 0.22 (0.07, 0.75) | **0.0182** | 0.09 |
| 282 | DG 16:1_18:1 | 0.39 (0.16, 0.97) | **0.0482** | 0.13 |
| 283 | DG 18:0_18:1 | 0.21 (0.06, 0.68) | **0.0119** | 0.08 |
| 284 | DG 18:0_18:2 | 0.26 (0.10, 0.73) | **0.0130** | 0.08 |
| 285 | DG 18:0_20:4 | 0.43 (0.19, 1.01) | 0.06 | 0.14 |
| 286 | DG 18:1_18:1 | 0.34 (0.13, 0.91) | **0.0367** | 0.11 |
| 287 | DG 18:1_18:2 | 0.36 (0.12, 1.09) | 0.08 | 0.17 |
| 288 | DG 18:1_18:3 | 0.33 (0.11, 1.01) | 0.06 | 0.14 |
| 289 | DG 18:1_20:3 | 0.86 (0.43, 1.71) | 0.66 | 0.75 |
| 290 | DG 18:1_20:4 | 0.54 (0.25, 1.20) | 0.14 | 0.25 |
| 291 | DG 18:2_18:2 | 0.62 (0.28, 1.37) | 0.24 | 0.37 |
| 292 | TG 14:0_16:0_18:1 | 0.48 (0.23, 0.99) | 0.05 | 0.14 |
| 293 | TG 14:0_16:0_18:2 | 0.57 (0.31, 1.04) | 0.07 | 0.17 |
| 294 | TG 14:0_16:1_18:1 | 0.40 (0.18, 0.86) | **0.0230** | 0.09 |
| 295 | TG 14:0_16:1_18:2 | 0.36 (0.15, 0.86) | **0.0251** | 0.10 |
| 296 | TG 14:0_17:0_18:1 | 0.56 (0.27, 1.12) | 0.11 | 0.22 |
| 297 | TG 14:0_18:0_18:1 | 0.32 (0.11, 0.88) | **0.0324** | 0.11 |
| 298 | TG 14:0_18:2_18:2 | 0.36 (0.15, 0.88) | **0.0297** | 0.11 |
| 299 | TG 14:1_16:0_18:1 | 0.58 (0.32, 1.05) | 0.08 | 0.17 |
| 300 | TG 14:1_16:1_18:0 | 0.54 (0.23, 1.25) | 0.16 | 0.28 |
| 301 | TG 14:1_18:0_18:2 | 0.52 (0.23, 1.17) | 0.12 | 0.23 |
| 302 | TG 14:1_18:1_18:1 | 0.44 (0.20, 0.97) | **0.0462** | 0.13 |
| 303 | TG 15:0_16:0_18:1 | 0.48 (0.21, 1.06) | 0.08 | 0.17 |
| 304 | TG 15:0_18:1_18:1 | 0.68 (0.39, 1.18) | 0.18 | 0.30 |
| 305 | TG 16:0_16:0_16:0 | 0.45 (0.20, 1.00) | 0.06 | 0.14 |
| 306 | TG 16:0_16:0_18:0 | 0.36 (0.14, 0.93) | **0.0392** | 0.12 |
| 307 | TG 16:0_16:0_18:1 | 0.50 (0.23, 1.07) | 0.08 | 0.17 |
| 308 | TG 16:0_16:0_18:2 | 0.44 (0.21, 0.96) | **0.0427** | 0.12 |
| 309 | TG 16:0_16:1_17:0 | 0.60 (0.32, 1.11) | 0.11 | 0.23 |
| 310 | TG 16:0_16:1_18:1 | 0.43 (0.19, 0.97) | **0.0475** | 0.13 |
| 311 | TG 16:0_17:0_18:0 | 0.50 (0.24, 1.06) | 0.08 | 0.17 |
| 312 | TG 16:0_17:0_18:1 | 0.56 (0.28, 1.13) | 0.11 | 0.23 |
| 313 | TG 16:0_17:0_18:2 | 0.61 (0.31, 1.20) | 0.16 | 0.28 |
| 314 | TG 16:0_18:0_18:1 | 0.23 (0.07, 0.79) | **0.0226** | 0.09 |
| 315 | TG 16:0_18:1_18:1 | 0.34 (0.14, 0.86) | **0.0259** | 0.10 |
| 316 | TG 16:0_18:1_18:2 | 0.40 (0.17, 0.91) | **0.0340** | 0.11 |
| 317 | TG 16:0_18:2_18:2 | 0.39 (0.16, 0.93) | **0.0380** | 0.11 |
| 318 | TG 16:1_16:1_16:1 | 0.61 (0.32, 1.19) | 0.15 | 0.27 |
| 319 | TG 16:1_16:1_18:0 | 0.32 (0.12, 0.82) | **0.0217** | 0.09 |
| 320 | TG 16:1_16:1_18:1 | 0.51 (0.25, 1.04) | 0.07 | 0.16 |
| 321 | TG 16:1_17:0_18:1 | 0.64 (0.34, 1.20) | 0.17 | 0.29 |
| 322 | TG 16:1_18:1_18:1 | 0.41 (0.17, 1.01) | 0.06 | 0.14 |
| 323 | TG 16:1_18:1_18:2 | 0.24 (0.07, 0.75) | **0.0176** | 0.09 |
| 324 | TG 17:0_18:1_18:1 | 0.39 (0.16, 0.96) | **0.0455** | 0.13 |
| 325 | TG 18:0_18:0_18:0 | 0.44 (0.21, 0.90) | **0.0299** | 0.11 |
| 326 | TG 18:0_18:0_18:1 | 0.23 (0.07, 0.69) | **0.0113** | 0.08 |
| 327 | TG 18:0_18:1_18:1 | 0.27 (0.10, 0.75) | **0.0143** | 0.08 |
| 328 | TG 18:0_18:2_18:2 | 0.32 (0.12, 0.88) | **0.0307** | 0.11 |
| 329 | TG 18:1_18:1_18:1 | 0.32 (0.12, 0.86) | **0.0281** | 0.10 |
| 330 | TG 18:1_18:1_18:2 | 0.39 (0.15, 0.97) | **0.0470** | 0.13 |
| 331 | TG 18:1_18:1_20:4 | 0.52 (0.22, 1.19) | 0.13 | 0.24 |
| 332 | TG 18:1_18:1_22:6 | 0.36 (0.15, 0.87) | **0.0279** | 0.10 |
| 333 | TG 18:1_18:2_18:2 | 0.35 (0.14, 0.89) | **0.0318** | 0.11 |
| 334 | TG 18:2_18:2_18:2 | 0.51 (0.24, 1.11) | 0.09 | 0.20 |
| 335 | TG 18:2_18:2_20:4 | 0.75 (0.35, 1.61) | 0.46 | 0.58 |

^1^ Cer d18:0 - dihydroceramide, Cer d18:1 - ceramide, HexCer - monohexocylceramide, Hex2Cer - dihexosylceramide, Hex3Cer - trihexosylceramide, GM3 - G_M3_ ganglioside, SM - sphingomyelin, PC - phosphatidylcholine, PC(O) - alkylphosphatidylcholine, PC(P) - alkenylphosphatidylcholine, LPC - lysophosphatidylcholine, LPC(O) - lysoalkylphosphatidylcholine, PE - phosphatidylethanolamine, PE(O) - alkylphosphatidylethanolamine, PE(P) – alkenylphosphatidylethanolamine, LPE - lysophosphatidylethanolamine, PI - phosphatidylinositol, LPI – lysophosphatidylinositol, PS - phosphatidylserine, PG - phosphatidylglycerol, BMP – bis monoacylglycerolphosphate, CE - cholesterol ester, ST 27:1/OH - cholesterol, DG - diacylglycerol and TG – triacylglycerol.

^2^ Odds ratio (95% confidence interval).

^3^ p-value based on logistic regression analysis adjusted for age and BMI.

^4^ p-values are corrected for multiple comparisons using the Benjamini-Hochberg method.
